# The use of communal rearing of families and DNA pooling in aquaculture genomic selection schemes

**DOI:** 10.1186/1297-9686-42-41

**Published:** 2010-11-22

**Authors:** Anna K Sonesson, Theo HE Meuwissen, Michael E Goddard

**Affiliations:** 1 Nofima Marin AS, Ås, Norway; 2 Department of Animal and Aquacultural Sciences, University of Life Sciences, Ås, Norway; 3 Department of Agriculture and Food Systems, University of Melbourne; 4 Victorian Department of Primary Industries, Australia

## Abstract

**Background:**

Traditional family-based aquaculture breeding programs, in which families are kept separately until individual tagging and most traits are measured on the sibs of the candidates, are costly and require a high level of reproductive control. The most widely used alternative is a selection scheme, where families are reared communally and the candidates are selected based on their own individual measurements of the traits under selection. However, in the latter selection schemes, inclusion of new traits depends on the availability of non-invasive techniques to measure the traits on selection candidates. This is a severe limitation of these schemes, especially for disease resistance and fillet quality traits.

**Methods:**

Here, we present a new selection scheme, which was validated using computer simulations comprising 100 families, among which 1, 10 or 100 were reared communally in groups. Pooling of the DNA from 2000, 20000 or 50000 test individuals with the highest and lowest phenotypes was used to estimate 500, 5000 or 10000 marker effects. One thousand or 2000 out of 20000 candidates were preselected for a growth-like trait. These pre-selected candidates were genotyped, and they were selected on their genome-wide breeding values for a trait that could not be measured on the candidates.

**Results:**

A high accuracy of selection, i.e. 0.60-0.88 was obtained with 20000-50000 test individuals but it was reduced when only 2000 test individuals were used. This shows the importance of having large numbers of phenotypic records to accurately estimate marker effects. The accuracy of selection decreased with increasing numbers of families per group.

**Conclusions:**

This new selection scheme combines communal rearing of families, pre-selection of candidates, DNA pooling and genomic selection and makes multi-trait selection possible in aquaculture selection schemes without keeping families separately until individual tagging is possible. The new scheme can also be used for other farmed species, for which the cost of genotyping test individuals may be high, e.g. if trait heritability is low.

## Background

Traditional family-based aquaculture breeding programs, in which families are kept separately until individual tagging and most traits are measured on the sibs of the candidates, are costly and require a high level of reproductive control, e.g. through stripping of the parents [1]. Therefore, alternatives to the above traditional family-based breeding programs are often used in aquaculture breeding schemes. The most widely used alternative is a selection scheme, in which families are reared communally and the candidates are selected based on their own individual measurements of the traits under selection. However, in the latter selection schemes, inclusion of additional traits depends on the availability of non-invasive techniques to measure the traits, such as the Torry Fat meter [2] to measure fat content, since family information is not available. This is a severe limitation of these schemes.

In genomic selection schemes [3], large numbers of (SNP) markers can be used instead of pedigree information and thus family-based selection schemes as in [4,5] are not needed. However, in aquaculture breeding there are many thousands of selection candidates and test individuals, which make genotyping costs high even if the genotyping costs per individual are low.

The aim of this paper is to develop a new selection scheme that combines communal rearing of families, pre-selection of candidates, DNA pooling and genomic selection and makes multi-trait selection possible in aquaculture selection schemes without keeping families separately until individual tagging is possible. We compare the effects of different designs on accuracy of selection, genetic gain and rates of inbreeding using computer simulations.

## Materials and methods

### Simulation of the starting population

A population with an effective population size (*N*_*e*_) of 1000 was simulated for 4000 generations according to the Fisher-Wright population model [6,7]. Five hundred males and 500 females were randomly selected and mated using sampling with replacement. From the last of these 4000 generations, generation zero (*G0*) of the selection population of the breeding scheme was obtained.

### Simulation of the breeding scheme in generations G0-G5

For generations *G0*-*G5*, the selection population was simulated as follows. One hundred sires and 100 dams (*Nfam *= 100) were randomly split into groups with *Nfampergroup *families per group (*Nfampergroup *= 1, 10 or 100, the latter resulting in all individuals being in one group). There was also one scheme with *Nfam *= 50 and *Nfampergroup *= 10. Each sire was randomly mated to one dam and vice versa, using sampling without replacement. Each mating resulted in a family that was split into one group of (*Ncand*/*Nfam*) selection candidates and a second group of (*Ntest*/*Nfam*) test individuals (*Ncand *= 20000 and *Ntest *= 2000, 20000 or 50000). Hence, family sizes were (*Ncand*+*Ntest*)/*Nfam *offspring with equal numbers of males and females. Every selection candidate family was grouped with (*Nfampergroup *- 1) other randomly chosen families. Similarly, every family of test individuals was grouped with the test individuals from the same (*Nfampergroup *- 1) other families as were the selection candidates, i.e. the same families were grouped together as test individuals and selection candidates. Strictly, it will not be necessary to group the candidate families separately, as classical parentage testing can be done using the same markers used to estimate the effects of the traits.

Two traits were considered: GROWTH, a trait measured on the *Ncand *selection candidates; and SIB_TRAIT, a trait that is measured on *Ntest *test individuals (sibs of the candidates), which were sacrificed to record the SIB_TRAIT.

The *Ncand *selection candidates were mass-selected across all families for their GROWTH phenotype. A total of *Npresel *candidates passed this preselection step, and (*Ncand*-*Npresel*) individuals were culled, *Npresel *being 1000 or 2000.

The test individuals were recorded for the SIB_TRAIT. Within each group, the 50% highest SIB_TRAIT individuals were sorted into the H-pool and the 50% lowest into the L-pool. DNA of the H-pool was extracted, pooled and genotyped. Similarly the L-pool's DNA was extracted, pooled and genotyped, which resulted in estimates of the within-pool frequencies of the marker alleles. These frequency estimates were assumed to contain no errors here. Marker effects were estimated and used to estimate the genome-wide breeding values (*GEBV*) for the SIB_TRAIT of the *Npresel *selection candidates (see Calculation of phenotypic values and true and estimated genome-wide breeding values). *Nfam *sires and *Nfam *dams were selected across families and groups from these preselected selection candidates using truncation selection for the SIB_TRAIT *GEBV*.

### Genome

Creation of the genomes of the population was as described in [4]. Briefly, the genome structure of individuals was diploid with 10 chromosomes 100 cM long. The infinite sites mutation model [8] was used to create new bi-allelic SNP, using a mutation rate of 10^-9 ^per nucleotide and assuming the number of nucleotides per cM to be 1000000. Inheritance of the SNP followed Mendel's law and the Haldane mapping function [9] was used to simulate recombinations. For each trait 50 SNP per chromosome were sampled randomly to be QTL (sampling without replacement from the SNP with minor allele frequency (MAF) >0.05). From the remaining SNP, 1000 with the highest MAF were chosen as genetic markers. This resulted in a total of 10000 markers spread over 1000 cM. Reduced numbers of markers were obtained by selecting every 10^th ^and 20^th ^marker, resulting in a number of markers, *Nmarkers *= 1000 and 500 markers, respectively. The reduced marker sets either reflected a situation where few markers are known or where genotyping costs are reduced by genotyping few markers.

Effects of the QTL alleles were sampled from the gamma distribution with a shape parameter of 0.4 and a scale parameter of 1.66 [10]. There were no pleiotropic QTL effects, and no genetic or environmental correlation between the two traits. The QTL effects were assumed to be either positive or negative with a probability of 0.5, because the gamma distribution only gives positive values. After sampling, these QTL allelic effects were standardized so that the total genetic variance was 1 for each trait.

### Calculation of phenotypic values and true and estimated genome-wide breeding values

The true genome-wide breeding value of an individual for *t *= GROWTH and *t *= SIB_TRAIT was calculated as:

$$TBV_{i}(t)=\sum_{j=1}^{500}x_{ij1}g_{j1}(t)+x_{ij2}g_{j2}(t).$$

where *x*_*ijk *_is the number of copies that individual *i *has at the *j*^th ^QTL position and *k*^th ^QTL allele, and *g*_*jk*_*(t) *is the effect of the *k*^th ^QTL allele at the *j*^th ^position. The phenotypic value of the individuals for trait *t *was simulated by adding an error term sampled from a normal distribution to the true breeding value (*TBV*_*i*_*(t))*:

$$P_{i}(t)=TBV_{i}(t)+\varepsilon_{i}(t)$$

where *ε_i_*(*t*) is an error term for animal *i*, which was normally distributed N(0, *σ*^*2*^_*e*_) and *σ*^*2*^_*e *_was adjusted so that the heritability was 0.4 for GROWTH and 0.1 or 0.4 for SIB_TRAIT.

The statistical model used to estimate the marker effects on SIB_TRAIT was the BLUP of marker effects method [3], using the mixed model equations:

(1) (X’X+Iλ)a=X’y

where ***a ***is a vector of the estimated SNP effects; ***X ***is a matrix of SNP genotypes, where element *X*_*ij *_equals the standardised genotype of individual *i *for SNP *j*, i.e. *X*_*ij *_is -2*p*_*j*_,/√*H*, (1-2*p*_*j*_)/√*H *or 2(1-*p*_*j*_)/√*H *for genotypes '11', '12', or '22', respectively, where *H *is heterozygosity (*H *= 2*p*_*j *_(1-*p*_*j*_)) and *p*_*j *_is allele frequency at locus *j*; *λ *is the variance ratio of the error variance to the SNP variance, which is the genetic variance divided by the number of SNP in the genome; *y*_*i *_is the phenotype of individual *i*, which is 1 (0) if *i *belongs to H (L)-pool. Thus, at this stage the phenotype is assumed binary, either because it is truly binary or because a continuous variable is split into two classes. Each pool (H, L) contains 50% of the individuals.

Since the test individuals are not individually genotyped, ***X***_***ij ***_is unknown, but ***X'X ***is expected to equal the (co)variance matrix of SNP genotypes (*X*_*ij*_) times the number of individuals (*n*). Here, the covariance matrix of the SNP genotypes will be estimated from the individually genotyped selection candidates instead of from the test individuals, i.e. element (*j,k*) of this matrix is calculated by Cov(*X*_*ij*_,*X*_*ik*_), where *X*_*ij *_is the standardised genotype of the *i*-th selection candidate.

Also ***X'y ***cannot be calculated because the test individuals are not individually genotyped. ***X'y ***is expected to equal the covariance between genotypes (*X*_*ij*_) and phenotypes times *n*. The following regression equation will be used to estimate the covariance between the genotypes and phenotypes:

$$\Delta x_{j}=b_{xj\;on\;y}*\Delta y$$

where *Δx*_*j *_is the average difference in allele frequency for SNP *j *between the individuals with '*y *= 1' and those with '*y *= 0'; *b*_*xj on y *_is the regression of the SNP genotype on the phenotype; and *Δy *is the difference in phenotype, which is 1. Since the variance of *y *is 0.25 (50% of the *y*'s are 1), the above regression equation reduces to:

$$\Delta x_{j}=Cov\left(X_{ij};y_{i}\right)/0.\text{25}$$

and thus *Cov*(*X*_*ij*_;*y*_*i*_) is estimated by 0.25* *Δx*_*j*_

where *Δx*_*j *_is recorded by the pooled genotyping of the '*y*_*i *_= 1' individuals and the '*y*_*i *_= 0' individuals. In conclusion, ***X'X ***is estimated by n*Cov(*X*_*ij*_*,X*_*ik*_) and ***X'y ***is estimated by n*Cov(X_*ij*_*,y*_*i*_*)*, which are needed for Equation [1], and Cov(*X*_*ij*_*,X*_*ik*_) and *Cov(X*_*ij*_*,y*_*i*_*) *are estimated from the genotypes of the selection candidates and from the pooled genotypes, respectively.

Estimated genome-wide breeding values for the selection candidates for SIB_TRAIT were obtained by summing the effects of the markers times the standardised genotypes times a regression coefficient to transform the GEBV from the binary data scale to the continuous data:

(2) $$GEBV_{i}(\text{SIBTRAIT})=b\sum_{j}^{n}X_{ij}a_{j}.$$

where the regression coefficient *b = Cov*(Σ *X_ij_a_j_*; *TBV_i_*)/*var*(Σ *X_ij_a_j_*), *TBV*_*i *_is the true breeding value of individual *i*. The regression *b *was calculated here using the the *TBV*_*i *_from the simulation. In practice, another method needs to be devised to estimate *b*, e.g. by regressing the phenotypes onto the EBV. This will reduce the selection accuracy, and this reduction depends on the available number of records to estimate the regression coefficient *b*. The regression coefficient *b *also corrects for the fact that genomic selection EBV may be biased in the sense that their variance is too big relative to that of the TBV [3].

Equation [2] implicitly incorporates the group means into the GEBV by using the estimates of the marker effects. In situations, where we have many continuously recorded phenotypes per group, the group means are expected to be more accurately estimated by the mean of the phenotypes of the individuals within the group. In this case, estimated genome-wide breeding values for the selection candidates for SIB_TRAIT were obtained by summing the effects of the markers within the group and adding a group-mean:

(3) $$GEBV_{i}(\text{SIBTRAIT})=b\left(\sum_{j}^{n}X_{ij}a_{j}-\mu_{GEBV}\right)+\mu_{p},$$

where *μ*_*p *_is the mean of the SIB_TRAIT-phenotypes of the individuals in group *p *to which individual *i *belongs; *μ_GEBV _*is the mean of the Σ *X_ij_a_j _*of all individuals in group *p*; and *b *is as in Equation [2].

In Equations [2] and [3], family means are implicitly estimated by the marker effects, as part of the total genetic effect. However, if *Nfampergroup *= 1, i.e. family means and group means coincide, the family means are estimated by the phenotypic averages of the group in Equation [3].

Selection of the candidates consisted of two steps: one pre-selection step, where selection was for GROWTH and one final selection step, where selection was for the SIB_TRAIT.

The accuracy of selection was calculated as the correlation between true and estimated breeding values among the pre-selected candidates for SIB_TRAIT (*acc*_*SIB_TRAIT*_). Inbreeding coefficients (*F*) were calculated based on pedigree, assuming that the *G0 *individuals were unrelated base parents.

### Statistics

Selection schemes were run for generations (*G0*-*G5*) and summary statistics for each of the schemes are based on 100 replicated simulations. The breeding schemes were compared for the accuracy of selection of the SIB_TRAIT (*acc*_*SIB_TRAIT*_), rate of inbreeding per generation (*ΔF*) and genetic gain of the SIB_TRAIT (*ΔG*_*SIB_TRAIT*_) and GROWTH (*ΔG*_*GROWTH*_), expressed in genetic standard deviation units of generation *G0 *(*σ*_*a*_)) in generation *G5*.

## Results

### Effect of number of markers, families per group and test individuals

Overall, there was an increase in accuracy of selection of the SIB_TRAIT (*acc*_*SIB_TRAIT*_) with an increasing number of markers especially when *Nmarkers *increased from 500 to 5000, but less so when it increased from 5000 to 10000 (Table 1). The *acc*_*SIB_TRAIT *_was lower with an increased number of families per group and the change in *acc*_*SIB_TRAIT *_was larger from *Nfampergroup *= 1 to *Nfampergroup *= 10 than from *Nfampergroup *= 10 to *Nfampergroup *= 100. *With Nfampergroup *= 1, the estimation of the family mean coincided with the estimation of the group mean such that the family mean was well estimated. With a higher number of families per group, only marker information was used to calculate family means (instead of phenotypic family means), which reduced *acc*_*SIB_TRAIT*_. This effect was larger with more families in the group.

**Table 1:** Results with different numbers of families per group, genetic markers and test individuals

**Nfampergroup**	**Nmarkers**	**acc**_**SIBTRAIT **_**(s.e.)**	**ΔF**	**ΔG**_**SIBTRAIT **_**(s.e.)**	**ΔG**_**GROWTH **_**(s.e.)**
		
			**Ntest = 2000**		
**1**	**500**	0.604 (0.005)	0.019	1.56 (0.03)	1.86 (0.03)
	**10000**	0.664 (0.004)	0.017	1.75 (0.02)	1.78 (0.03)
**10**	**500**	0.502 (0.007)	0.013	1.43 (0.04)	1.77 (0.03)
	**10000**	0.603 (0.004)	0.012	1.68 (0.03)	1.79 (0.03)
**100**	**500**	0.489 (0.006)	0.011	1.38 (0.04)	1.77 (0.03)
	**10000**	0.580 (0.005)	0.011	1.59 (0.02)	1.79 (0.02)
			**Ntest = 20000**		
**1**	**500**	0.723 (0.003)	0.013	1.87 (0.02)	1.84 (0.03)
	**5000**	0.838 (0.002)	0.011	2.10 (0.03)	1.85 (0.03)
	**10000**	0.848 (0.002)	0.013	2.06 (0.02)	1.81 (0.02)
**10**	**500**	0.608 (0.004)	0.013	1.68 (0.03)	1.73 (0.03)
	**5000**	0.802 (0.003)	0.010	2.03 (0.02)	1.72 (0.03)
	**10000**	0.817 (0.002)	0.012	2.06 (0.02)	1.80 (0.03)
**100**	**500**	0.600 (0.005)	0.013	1.63 (0.03)	1.72 (0.03)
	**5000**	0.789 (0.002)	0.011	2.00 (0.02)	1.74 (0.03)
	**10000**	0.808 (0.002)	0.011	2.05 (0.02)	1.81 (0.02)
			**Ntest = 50000**		
**1**	**500**	0.732 (0.004)	0.018	1.87 (0.03)	1.84 (0.03)
	**10000**	0.877 (0.002)	0.012	2.09 (0.02)	1.78 (0.02)
**10**	**500**	0.630 (0.005)	0.013	1.69 (0.03)	1.70 (0.03)
	**10000**	0.850 (0.002)	0.009	2.10 (0.02)	1.78 (0.03)
**100**	**500**	0.609 (0.005)	0.012	1.65 (0.03)	1.74 (0.03)
	**10000**	0.845 (0.002)	0.011	2.10 (0.02)	1.83 (0.02)

Accuracy of selection of the SIB_TRAIT (*acc*_*SIB_TRAIT*_), rates of inbreeding (*ΔF*) and genetic gain of the SIB_TRAIT (*ΔG*_*SIB_TRAIT*_) and GROWTH (*ΔG*_*GROWTH*_) in generation G5 with different numbers of families per group (*Nfampergroup*), test individuals (*Ntest*) and markers (*Nmarkers*). The heritability of the SIB_TRAIT was 0.4, number of families (*Nfam*) was 100 and the number of preselected candidates (*Npresel*) was 1000. s.e. of ΔF was between 0.001 and 0.002

With a lower number of test individuals, i.e. *Ntest *= 2000, *acc*_*SIB_TRAIT *_was much lower than with larger numbers of test individuals. With the largest numbers of markers, i.e. *Nmarkers *= 10000, *acc*_*SIB_TRAIT *_was only 0.664, 0.603 and 0.580, respectively, for *Nfampergroup *= 1, 10 and 100. The difference in *acc*_*SIB_TRAIT *_between *Ntest *= 20000 and 50000 was small. With *Ntest *= 50000 and *Nmarkers *= 10000, *acc*_*SIB_TRAIT *_was 0.877, 0.850 and 0.845, respectively for *Nfampergroup *= 1, 10 and 100, and thus depended little on *Nfampergroup *in this case, which indicates that family means were accurately estimated by the markers with such high numbers of test individuals. The latter scheme was the scheme with the overall highest *acc*_*SIB_TRAIT*_.

The genetic gain for the SIB_TRAIT (*ΔG*_*SIB_TRAIT*_) corresponded well to the patterns of changes in *acc*_*SIB_TRAIT*_. The genetic gain for GROWTH (*ΔG*_*GROWTH*_) did not vary much between the schemes, except that *ΔG*_*GROWTH *_was somewhat increased with *Nfampergroup *= 1 and low marker density.

Overall, rates of inbreeding (*ΔF*) did not differ much between the schemes except that there was a tendency for a higher *ΔF *with *Nfampergroup *= 1 than with 10 or 100. With *Nfampergroup *= 10 or 100, markers are used to estimate family means, which may result in reduced estimates of between-family differences, and thus relatively more within-family selection. There was also a small tendency for higher ΔF with *Nmarkers *= 500 than *Nmarkers *= 10000.

### Effect of heritability of SIB_TRAIT

With a lower heritability of the SIB_TRAIT, i.e. 0.1, accuracy of selection was reduced, as expected (Table 2). However, *acc*_*SIB_TRAIT *_was still rather high with a large *Ntest*. For example, with *Nfampergroup *= 10 and *Ntest *= 20000 and 50000, *acc*_*SIB_TRAIT *_was 0.557 and 0.701, respectively, for *Nmarkers *= 500 only. The effect of heritability on *acc*_*SIB_TRAIT *_was smallest for the scheme with *Ntest *= 50000.

**Table 2:** Results with reduced heritability of the SIB_TRAIT

**Nfampergroup**	**Nmarkers**	**acc**_**SIBTRAIT **_**(s.e.)**	**ΔF**	**ΔG**_**SIBTRAIT**_**(s.e.)**	**ΔG**_**GROWTH**_**(s.e.)**
		
			**Ntest = 2000**		
**1**	**500**	0.457 (0.001)	0.021	1.25 (0.04)	1.91 (0.03)
	**10000**	0.490 (0.001)	0.020	1.35 (0.03)	1.84 (0.03)
**10**	**500**	0.356 (0.007)	0.012	1.07 (0.03)	1.80 (0.03)
	**10000**	0.405 (0.005)	0.010	1.19 (0.03)	1.79 (0.03)
			**Ntest = 20000**		
**1**	**500**	0.667 (0.005)	0.017	1.74 (0.03)	1.83 (0.03)
	**10000**	0.739 (0.003)	0.015	1.89 (0.02)	1.84 (0.03)
**10**	**500**	0.557 (0.006)	0.012	1.54 (0.03)	1.78 (0.03)
	**10000**	0.693 (0.004)	0.012	1.84 (0.02)	1.82 (0.03)
			**Ntest = 50000**		
**1**	**500**	0.701 (0.004)	0.017	1.81 (0.03)	1.87 (0.03)
	**10000**	0.813 (0.003)	0.014	2.06 (0.03)	1.84 (0.03)
**10**	**500**	0.596 (0.005)	0.014	1.63 (0.03)	1.75 (0.03)
	**10000**	0.780 (0.003)	0.012	2.06 (0.03)	1.78 (0.03)

The heritability of the SIB_TRAIT was 0.1, *Nfam *was 100 and Npresel was 1000. s.e. of ΔF was between 0.001 and 0.002

Overall, genetic gain for the SIB-TRAIT (*ΔG*_*SIB_TRAIT*_) followed the pattern of changes of the accuracy of selection. The genetic gains for GROWTH (*ΔG*_*GROWTH*_) were generally higher than in Table 1, which is probably due to the lower selection pressure on the SIB_TRAIT when the heritability is reduced. The reduced selection pressure for the SIB_TRAIT results in smaller allele frequency changes of QTL affecting the SIB_TRAIT and of linked positions in the genome. The reduced frequency changes/genetic drift at linked positions implies that the selection pressure for GROWTH results in more response for GROWTH. Rates of inbreeding (*ΔF*) were somewhat higher than with a higher heritability of the SIB-TRAIT, i.e. 0.4, but showed a similar pattern across the schemes. The ΔF is not much affected by the heritability of the SIB_TRAIT, because selection for the SIB_TRAIT is not based on phenotypes but on marker genotypes.

### Effect of preselection and number of families

There was little difference in accuracy of selection with *Npresel *= 1000 or 2000 (Table 3). For *Nmarkers *= 500, 5000 or 10000, *acc*_*SIB_TRAIT *_was 0.608, 0.802 and 0.817, respectively, with *Npresel *= 1000, and 0.635, 0.792 and 0.803, respectively, with *Npresel *= 2000.

With *Nfam *= 50 instead of 100, *acc*_*SIB_TRAIT *_increased somewhat due to the larger full-sib family sizes, and was 0.694, 0.825 and 0.837 for *Nmarkers *= 500, 5000 and 10000.

*ΔF *was as expected much more increased with *Nfam *= 50 than with *Nfam *= 100. For example, with *Nmarkers *= 5000, *ΔF *increased from 0.010 to 0.020 when *Nfam *decreased from 100 to 50 (Table 3).

**Table 3:** Results with different numbers of pre-selected candidates and families

**Nmarkers**	**acc**_**SIBTRAIT **_**(s.e.)**	**ΔF**	**ΔG**_**SIBTRAIT **_**(s.e.)**	**ΔG**_**GROWTH **_**(s.e.)**
	
	**Nfam = 100**		**Npresel = 1000**	
**500**	0.608 (0.004)	0.013	1.68 (0.03)	1.73 (0.03)
**5000**	0.802 (0.003)	0.010	2.03 (0.02)	1.72 (0.03)
**10000**	0.817 (0.002)	0.011	2.06 (0.02)	1.80 (0.03)
	**Nfam = 100**		**Npresel = 2000**	
**500**	0.635 (0.005)	0.018	2.14 (0.04)	1.29 (0.03)
**5000**	0.792 (0.002)	0.013	2.45 (0.03)	1.34 (0.02)
**10000**	0.803 (0.002)	0.012	2.48 (0.03)	1.32 (0.02)
	**Nfam = 50**		**Npresel = 1000**	
**500**	0.694 (0.004)	0.029	2.38 (0.04)	1.08 (0.04)
**5000**	0.825 (0.002)	0.020	2.78 (0.04)	1.20 (0.04)
**10000**	0.837 (0.002)	0.022	2.85 (0.03)	1.24 (0.03)

The heritability of the SIB_TRAIT was 0.4, *Ntest *was 20000 and *Nfampergroup *was 10. s.e. of ΔF was between 0.001 and 0.003

### Group means estimated from genetic markers instead of phenotypes

Table 4 shows the results with the same parameters as in Table 1, but where group means of the selection candidates were estimated using genetic markers instead of phenotypic means. The latter may be necessary when common environmental group effects occur meaning that the phenotypic group means are not representative of the genetic mean of the group. In general, Table 4 shows an increasing trend for *acc*_*SIB_TRAIT *_with increasing *Nmarkers*, especially from *Nmarkers *= 500 to 5000. It also shows that the *acc*_*SIB_TRAIT *_was much lower with *Nfampergroup *= 1 than with *Nfampergroup *= 10 and 100, because the family effect cannot be well estimated by the markers since the group and family means are confounded in case of *Nfampergroup *= 1.

**Table 4:** Results with genetic markers to estimate group means

**Nfampergroup**	**Nmarkers**	**acc**_**SIBTRAIT **_**(s.e.)**	**ΔF**	**ΔG**_**SIBTRAIT **_**(s.e.)**	**ΔG**_**GROWTH **_**(s.e.)**
	
			**Ntest = 2000**		
**1**	**500**	0.290 (0.007)	0.008	0.89 (0.03)	1.86 (0.03)
	**10000**	0.403 (0.005)	0.006	1.18 (0.02)	1.77 (0.02)
**10**	**500**	0.483 (0.006)	0.014	1.39 (0.03)	1.78 (0.02)
	**10000**	0.586 (0.004)	0.011	1.64 (0.03)	1.76 (0.02)
**100**	**500**	0.489 (0.006)	0.011	1.38 (0.04)	1.77 (0.03)
	**10000**	0.580 (0.004)	0.011	1.59 (0.02)	1.79 (0.02)
			**Ntest = 20000**		
**1**	**500**	0.373 (0.006)	0.006	1.14 (0.03)	1.79 (0.03)
	**5000**	0.610 (0.005)	0.007	1.75 (0.02)	1.86 (0.02)
	**10000**	0.642 (0.004)	0.006	1.81 (0.02)	1.85 (0.02)
**10**	**500**	0.608 (0.005)	0.014	1.67 (0.03)	1.70 (0.03)
	**5000**	0.788 (0.002)	0.010	2.03 (0.02)	1.79 (0.03)
	**10000**	0.810 (0.002)	0.013	2.08 (0.03)	1.80 (0.03)
**100**	**500**	0.600 (0.005)	0.013	1.63 (0.02)	1.72 (0.03)
	**5000**	0.790 (0.002)	0.011	2.00 (0.02)	1.75 (0.03)
	**10000**	0.808 (0.002)	0.012	2.04 (0.02)	1.80 (0.02)
			**Ntest = 50000**		
**1**	**500**	0.393 (0.006)	0.008	1.21 (0.03)	1.83 (0.02)
	**10000**	0.673 (0.005)	0.006	1.89 (0.02)	1.83 (0.03)
**10**	**500**	0.616 (0.005)	0.014	1.71 (0.03)	1.76 (0.02)
	**10000**	0.841 (0.002)	0.010	2.11 (0.02)	1.79 (0.02)
**100**	**500**	0.609 (0.005)	0.012	1.65 (0.03)	1.74 (0.03)
	**10000**	0.845 (0.002)	0.011	2.10 (0.02)	1.82 (0.02)

Variables are as in Table 1, i.e. the heritability of the SIB_TRAIT was 0.4, *Nfam *was 100 and *Npresel *was 1000. s.e. of ΔF was 0.001

The *ΔF *increased when *Nfampergroup *increased from 1 to 10, but not from 10 to 100, e.g. with *Ntest *= 20000 and *Nmarkers *= 5000, *ΔF *was 0.007 with *Nfampergroup *= 1 and 0.013 with *Nfampergroup *= 10. With *Nfampergroup *= 1, markers cannot estimate the family means, in which case selection is for within-family deviations as estimated by the markers, i.e. within-family selection, which is known to result in low rates of inbreeding.

When comparing Tables 1 and 4, *acc*_*SIB_TRAIT *_depends highly on *Nfampergroup*. If *Nfampergroup = *1, *acc*_*SIB_TRAIT *_was considerably lower when the family means were estimated by markers rather than by phenotypic values only, e.g. 0.610 (Table 4) compared to 0.838 (Table 1) with *Ntest *= 20000. If *Nfampergroup *= 10, *acc*_*SIB_TRAIT *_was only somewhat lower when family means were estimated using markers and if *Nfampergroup=*100, *acc*_*SIB_TRAIT *_was equal for both methods. Hence, markers are increasingly more efficient in estimating family effects with increasing *Nfampergroup*.

## Discussion

Implementation of genomic selection in aquaculture breeding schemes is hampered by the large number of individuals that need to be genotyped [4]. Here, we present a method to apply DNA pooling in genomic selection, which dramatically reduces the genotyping costs of the test-population [11]. The DNA pooling further avoids pedigree recording, as is the case in traditional family-based designs, in the test-population, and the dense SNP genotyping also achieves this in the selection candidate groups. In addition, the low genotyping costs of the DNA pools make it very cost-effective to extend the test group to more traits that can only be measured on sibs of the candidates, i.e. towards highly multitrait breeding schemes. A methodology to estimate SNP effects from DNA pooling data was derived and yielded high selection accuracies, i.e. 0.60-0.85 with a large number of test individuals. This was especially the case if *Ntest *= 20000 or more for the aquaculture breeding schemes used here, even when multiple families were grouped and genotyping of pooled samples was done. The accuracy of selection decreased with an increasing number of families per group. If *Ntest *was only 2000, selection accuracy was substantially reduced, showing the importance of having large numbers of phenotypic records to accurately estimate marker effects.

The methodology presented here for DNA pooling in genomic selection will be beneficial to most species, where genomic selection is applied. In most species, the cost of genotyping large numbers of test individuals hampers seriously implementation of genomic selection. Genomic selection is currently mostly used in dairy cattle, where the use of accurately progeny tested bulls reduces the size of the test population. Still, Van Raden et al. [12] have had to genotype 3600 test bulls to obtain a high selection accuracy. Furthermore, the use of genomic selection instead of progeny testing for the selection of bulls implies that there will be no progeny tested bulls available in future dairy cattle schemes. Thus, in the future, the test population will consist of very large numbers of phenotypically recorded cows and the presented DNA pooling strategies can greatly reduce the genotyping costs even in dairy cattle by pooling DNA samples from cows with high and low phenotypic values, instead of individually genotyping the large numbers of cows.

Selection accuracy of these schemes can be compared to a family-based genomic selection breeding program. For example, Nielsen et al. [5] have reported selection accuracies of about 0.8 for a breeding program with 2000 test individuals, a trait with a 0.4 heritability and 100 families. Their scheme can be compared to the results of Table 1, which shows that *Ntest *= 2000 has a selection accuracy of about 0.60-0.65. Hence, the schemes with *Ntest *= 2000 have a selection accuracy 0.20-0.25 lower with genotyping of pooled samples than with genotyping of all individuals. However, *acc*_*SIB_TRAIT *_was approximately the same as for the larger *Ntest *= 20000 or 50000 here with *acc*_*SIB_TRAIT *_of 0.60-0.85 and 0.60-0.90, respectively.

Genetic gain for GROWTH was increased in Table 1 when *Nfampergroup *= 1 and marker density was low. In this situation, the estimation of the marker effects resembles that of a TDT (Transmission Disequilibrium Test) for quantitative traits, where the effect of the marker is also estimated within families but is expected to be the same across all families, i.e. the markers are picking-up LD but are corrected for family effects (spurious associations). If the marker density is low, the markers will show only low LD with the QTL, and since they are also not picking up family effects, marker effects will be small. The latter results in a relatively low efficiency of the marker-assisted selection part of the selection for SIB_TRAIT and thus in relatively small allele frequency changes of positions linked to the largest SIB_TRAIT QTL. The latter implies that the selection for GROWTH is not hindered by such frequency changes and thus may explain why the selection for GROWTH is relatively efficient when *Nfampergroup *= 1 and marker density is low.

We also investigated the effect of different correlations between GROWTH and SIB_TRAIT. Here we assumed that every QTL had correlated multi-normally distributed effects for GROWTH and SIB_TRAIT with a correlation of 0.3, 0.0 and -0.3 (since we lacked a Multitrait-Laplacian distribution sampler). With group means estimated as the mean of the phenotypes of the individuals within the group and *Nfamperpool *= 10, *ΔG*_*GROWTH *_was reduced by 18% and *ΔG*_*SIB_TRAIT *_by 24% when the correlation was -0.3 instead of 0.0. With a correlation of 0.3, *ΔG*_*GROWTH *_increased by 20% and *ΔG*_*SIB_TRAIT *_by 24%.

The breeding scheme suggested here relies heavily on the success of genotyping pooled samples. Our method assumed accurately estimated allele frequencies in both the L- and H-pools, but estimation errors on the pool mean frequencies have been reported, e.g. variance of the estimation error, i.e. the so-called technical error was estimated by Craig et al. [13] to be 6.8 × 10^-5^. Macgregor et al. [11] have reported that these errors depends on several parameters, such as density of the SNP chip, pooling strategy and array dependent parameters such as number of beadscores per SNP. Baranski et al. [14] have found a correlation between individual and pooled genotypes of 0.98 for a scheme with 60 families, one animal/family/pool, and three replicates per pool. Each pool consisted of susceptible and resistant groups for infectious salmon anemia of Atlantic salmon, where 15 individuals per family had been individually tested for the disease.

The improved results with large numbers of test fish per pool suggest that the accurate estimation of allele frequencies in the high and low pool are crucial to estimate the marker effects. In case the DNA pooling technique does not achieve such a high accuracy, the DNA pooling can be replicated in order to achieve the required accuracy, i.e. the error variance of the average of the allele frequencies estimates over all 'low' ('high') replicated pools is *p*(1-*p*)/*N *+ *V*_*t*_/*m*, where *p *is the true allele frequency, *N *is the total number of individuals in all 'low' ('high') pools, *m *is the number of replicated DNA poolings, and *V*_*t *_is the technical error due to the pooling technique, which we assumed to equal 0. The *V*_*t*_/*m *term can be reduced by increasing the number of replicates. Our numbers of individuals of 2,000/2, 20,000/2 and 50,000/2 could be interpreted as an effective numbers of individuals, *N*_*e*_, where

$$N_{e}={\left[\text{1}/N+V_{t}/\left(p\left(\text{1}-p\right)m\right)\right]}^{-\text{1}}.$$

Given this equation for *N*_*e*_, combinations of *N*, *m*, *V*_*t *_and *p *can be found that result in error variances similar to those presented in this paper.

Selection accuracy for quantitative traits may be further improved by removing individuals around the population mean from the DNA pools, which will increase the differences in allele frequencies. However, the number of individuals within each of the DNA pools will be reduced, which increases the variability of the allele frequency estimates. The former will improve selection accuracy whilst the latter will reduce it. Thus, further research is needed to investigate the optimal phenotypic selection differential between the two DNA pools.

The genotyping costs of the test individuals have been much reduced by the grouping strategy. However, we still require genotyping of the selection candidates. Due to the preselection step for GROWTH, the number of candidates to be genotyped was reduced from 20000 to 1000 or 2000 in this scheme, which hardly affected the *acc*_*SIB_TRAIT*_. Hence, there will still be a considerable number of individuals to be genotyped. The costs of this genotyping could be reduced by applying a low-density SNP chip to these candidates, as suggested by Habier et al. [15].

The grouping strategy may help to correct for the skewed contribution of parents that often occurs in mass spawning populations, see e.g. [16]. The number of families that should be reared per group to reduce the skewedness of parental contributions needs to be optimised per population.

Phenotyping 20000 animals for the sib trait might be very costly but that will depend on the trait. For instance, if the trait was resistance to a disease challenge, the phenotyping might simply consist in sorting the dead and alive fish.

## Conclusions

This new selection scheme combines communal rearing of families, pre-selection of candidates, DNA pooling and genomic selection and makes multi-trait selection possible in aquaculture selection schemes without keeping families separately until individual tagging is possible. The new scheme can also be used for other farmed species, for which the cost of genotyping test individuals may be high, e.g. if trait heritability is low.

## Competing interests

The authors declare that they have no competing interests.

## Authors' contributions

AKS wrote the main computer program, ran computer programs and drafted the manuscript. MEG developed method for estimating SNP effects using pooled DNA data. THEM wrote computer modules for genome-wide breeding value estimation and for Fisher-Wright populations. All authors have approved the final manuscript.
